# Comparison of Maturity in Auditory-Visual Multisensory Processes With Sound-Induced Flash Illusion Test in Children and Adults

**DOI:** 10.7759/cureus.27631

**Published:** 2022-08-03

**Authors:** Kiana Kheirkhah, Vahid Moradi, Iman Kavianpour, Saeid Farahani

**Affiliations:** 1 Department of ENT, Sint-Augustinus Antwerp, European Institute For Otorhinolaryngology (ORL), Antwerp, BEL; 2 Department of Audiology, School of Rehabilitation Sciences, Tehran University of Medical Sciences, Tehran, IRN; 3 Department of Telecommunication, School of Engineering Boushehr Branch, Islamic Azad University, Boushehr, IRN

**Keywords:** normal hearing subjects, behavioral test, sound-induced flash illusion, multi-sensory neural maturation, auditory visual integration

## Abstract

Multi-sensory neural pathways of auditory-visual maturation develop before birth in humans. Maturation levels of multi-sensory brain pathways are very different in children in comparison to adults. Auditory sensory neural maturation occurs earlier than visual sensory neural maturation. Auditory-visual multi-sensory processing can assess using objective (event-related potential tests) and subjective (behavioral tests). In this study, we use the sound-induced flash illusion (SIFI) test as a subjective test. The results of the SIFI test show that when the test is complex, the younger children performed not as accurately as the older ones. This difference occurs due to the complexity in the auditory-visual multi-sensory pathway maturation. Therefore, it is necessary to evaluate and analyze the results of different age groups with their normative data.

## Introduction

Before birth, all sensory, auditory, and somatosensory sensations begin to gather information and send it to the cortex. The synaptic density gradually increases [1], and the brain volume grows up by four-fold [2]. All these changes lead to the maturation of the different senses’ nerves. In unisensory mode, each sense transmits information to its specific brain region. For instance, the auditory signal stimulates the cochlea and produces a firing rate in the eighth cranial nerve, and this information eventually reaches the temporal cortex through the brainstem and midbrain [3]. In that case, it will ultimately affect the sophisticated behavioral, perceptual, and emotive processes and lead to better understanding and more comprehensive decision-making [4].

In hearing-impaired children, for example, when hearing loss is profound, and hearing aids cannot provide proper amplification, children use the visual sense. Through lip-reading, they compensate for hearing loss. Consequently, discrimination and understanding of the spoken sentence are improved [5]. Two factors, auditory and visual sensations, are responsible for the maturation of multi-sensory neural pathways. The different senses of infants gather information, gain more experience, and reach maturity in adolescence. The information from the cortex is essential because the cortex controls the function of the neural pathways located in the brainstem and midbrain [6]. Thus, if either of these two factors is affected, the maturity of the multi-sensory processing will be affected. Very different maturation levels of multi-sensory brain pathways were observed in comparisons of children with severe to profound hearing loss and children of a similar age with normal hearing [7]. If cochlear implantation is provided for children with severe to profound hearing loss, the auditory pathways gather information, and neural maturity will be complete [8]. The second factor is cortical health. If cortex input to the lower regions is restricted, the maturation of the multi-sensory neural pathways will be affected. For example, in autistic children, because the cortex is affected, it cannot control the midbrain and brainstem inputs. Thus, the maturation of the auditory-visual neural pathways is not complete [9].

Auditory and visual inputs enter the brainstem and harmonize in the superior colliculus (SC) nucleus [10]. Finally, the neural message from the SC reaches the anterior ectosylvian sulcus region located in the cortex, and it shares information with the temporal, occipital, parietal, and prefrontal cortex [11]. Neural maturation time varies for different senses. Neural maturation of auditory pathways occurs earlier for auditory-visual multi-sensory processing than for visual pathways [12]. The maturation of different regions of the brain also differs. For example, midbrain maturation occurs before cortex maturation [6]. Therefore, in assessing and comparing people of different ages, especially at younger ages, neural maturation should be considered; otherwise, test results will be severely affected.

Auditory-visual multi-sensory processing can be analyzed using an objective method, such as event-related potential (ERP) tests [13] or through subjective and behavioral tests [14]. Many studies have been done in the field of electrophysiological tests. Brandwein et al. in 2011 studied ERP responses in three age groups of children (7-9, 10-12, and 13-16 years) [13]. A monitor was placed in front of the participants in a dimly-lit room. The participants were asked to focus on the monitor to minimize the movement artifacts. When the participants saw the red circle, heard the tone, or saw the circle and also heard the tone, they were instructed to press a button on the keypad as quickly as possible for all three stimuli types (Auditory Alone, Visual Alone, and Auditory and Visual Simultaneous) in blocks of 100 trials. According to a uniform distribution, the interstimulus interval varied randomly from 1 to 3 (s) while breaks were encouraged between blocks to reduce fatigue, and stickers and verbal praise were provided for children. Their results show that the latency of P1 and N1 waves decreases with age and the amplitude of the waves increases. These changes reflect the maturation of the neural pathways. The comparison of ERP waves of children in three age groups (7-9, 10-12, and 13-16 years) with adults revealed that brain maturity in a 15-year-old adolescent is similar to that of an adult. Therefore, it can be suggested that multi-sensory maturity is complete at 15-year-old [13].

A sound-induced flash illusion (SIFI) indicates that while a single visual flash is presented by various auditory beeps, the number of perceived flashes differs from the real presented flash. The effect of SIFI on different age groups is the aim of this research.

This study analyzed auditory-visual multisensory processing through behavioral testing to determine whether the results of behavioral tests are consistent with the results of electrophysiological tests based on the recorded data. To this end, the SIFI test was employed in this research, which evaluates auditory-visual multi-sensory processing. The test was performed on children of different age groups and compared with the adults’ performance.

## Materials and methods

All volunteers were selected from the Najva Hearing Center, Tehran, Iran. The participating children and adolescents were divided into three groups: children 7-9 years old (n=15; eight females; mean age 7.67 years), 10-12 years old (n=15; eight females; mean age 10.93 years), and adolescents 13-16 years old (n=15; seven females; mean age: 14.93 years). Adults formed the fourth group (n=15; eight females; mean age 33 years). All participants were assessed by audiometry (250 Hz to 8000 Hz frequency) and tympanometry. Normal hearing thresholds (0-20 dBHL) in all frequencies and the health of the tympanic membrane and middle ear were confirmed in both ears [15]. the vision of all participants was evaluated based on the Snellen chart to ensure visual health [16]. The participants had no history of mental illness, brain diseases, head trauma, or consumption of psychiatric drugs [17]. Written consent information was obtained from all participants. The study was approved by the Human Research Ethics Committee of Tehran University of Medical Sciences (IR.TUMS.FNM.REC.1398.139).

**Figure 1:**
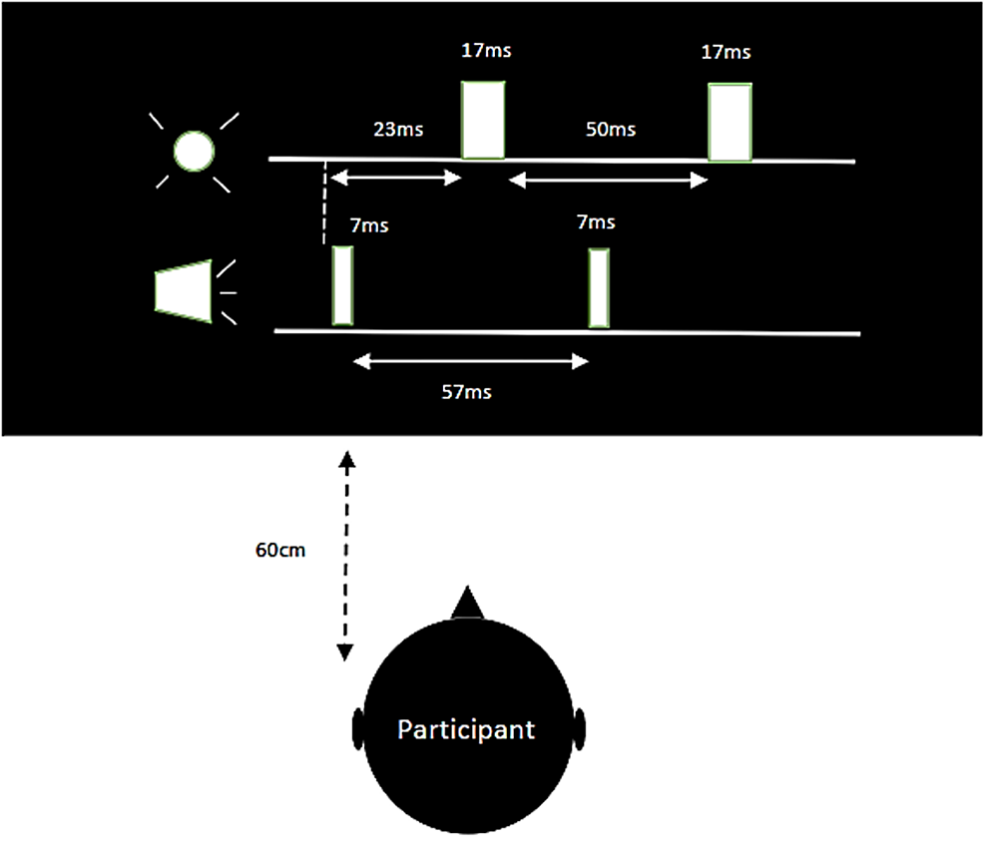
Temporal profile of the flash and beep stimuli in SIFI test SIFI: sound-induced flash illusion

All parameters and measures needed to perform the SIFI test were taken from Shams et al. [18]. To perform the SIFI test, a beep with a 3500 Hz frequency and duration of 7 ms at 95 dB sound pressure level (SPL) by two speakers on either side of the screen was presented. The interval between the beeps was 57 ms. The beeps always provided 23 ms before the flashes, which were provided with a duration of 17 ms and luminescence of 108 cd/m2 at an angle of 2-5 degrees of visual field eccentricity on a black laptop screen (model ASUS X550, ASUSTek Computer Inc., Beitou District, Taipei, Taiwan) with a luminescence of 0.02 cd/m2. The interval between flashes was 50 ms [18]. During the test, volunteers sat on a chair 60 cm away from the laptop screen and were asked to continuously focus on the screen and state the number of flashes they saw. The test was conducted in a quiet, dimly-lit room, and volunteers were asked to turn off their cellphones. Details are shown in Figure 1.

In the SIFI test, a flash was perceived along with 0-4 beeps. The participants were asked to indicate the number of flashes they could observe while tests presented interleaved five times for each participant; a total of 100 trials were performed. The results of four groups were recorded and then analyzed. All analyses were performed by IBM SPSS Statistics for Windows, Version 22.0 (Released 2013; IBM Corp., Armonk, New York, United States). Analysis of variance (ANOVA) was used to compare the mean number of flashes perceived by each age group. A level of less than 0.05 was considered significant.

## Results

The same results were observed in all four groups when the flash was presented with zero and/or one beep, and no significant difference was observed. However, increasing the number of beeps from two to four allocated different results, and there was great variability between the groups. Table 1 shows each group's average score of perceived flashes for 0-4 beeps while only one flash is presented.

**Table 1:** Mean and standard deviation of perceived flashes by each group and presented modes F: flashes; B: beeps; p: P-value

Age groups			F1B0	F1B1	F1B2	F1B3	F1B4
7-9 years		Mean	1.00	1.00	1.40	2.13	2.33
		Std. Deviation	.000	.000	.507	.352	.488
10-12 years		Mean	1.00	1.00	1.60	2.67	3.07
		Std. Deviation	.000	.000	.507	.488	.704
13-16 years		Mean	1.00	1.00	1.93	2.93	3.33
		Std. Deviation	.000	.000	.258	.258	.488
Adult		Mean	1.00	1.00	2.00	2.87	3.33
		Std. Deviation	.000	.000	.000	.516	.488
ANOVA statistics		Between Groups	p= 1.000	p= 1.000	p= 0.000	p= 0.000	p= .000

As illustrated in Table 1, when a flash presented with zero beeps (F1B0) or one beep (F1B1), the perceived number of flashes was quite similar in all groups, indicating that auditory stimuli could not influence the perception of visual stimuli. However, completely different results were achieved when the number of beeps was increased from two to four. As the age of the participants increased, the mean number of perceived flashes also increased. In the F1B2 test, the number of perceived flashes for the age group of 7-9 years equaled 1.40. In adults, the number of perceived flashes was two, which means that when a flash and two beeps were presented, participants perceived two flashes. This phenomenon is called visual illusions and illustrates the combined effect of auditory and visual information. In the F1B2, F1B3, and F1B4 tests, the results of perceived flashes for the 7-9 years age group differed significantly from those for adults due to the effects of maturation of auditory-visual multi-sensory processing. These differences decreased with age.

Tukey's HSD (honestly significant difference) analysis was used to compare the differences between the groups in different modes of visual-auditory stimulation. The results are shown in Table 2. Because there was no difference between the groups in the F1B0 and F1B1 tests, Table 2 shows the results for F1B2, F1B3, and F1B4.

**Table 2:** Comparison of differences between groups in different modes of visual-auditory stimulation with Tukey's HSD test. *. The mean difference is significant at the 0.05 level HSD: honestly significant difference

Test	Group (I)	Group (J)	Mean Difference (I-J)	Sig.	
					F1B2	Adult	7-9 years	.600	0.000*	
10-12 years	.400	0.028*				
13-16 years	.067	0.963				
7-9 years	Adult	-.600	0.000*		
	10-12 years	-.200	0.482		
	13-16 years	-.533	0.002*		
10-12 years	Adult	-.400	0.028*		
	7-9 years	.200	0.482		
	13-16 years	-.333	0.090		
13-16 years	Adult	-.067	0.963		
	7-9 years	.533	0.002*		
	10-12 years	.333	0.090		
F1B3	Adult	7-9 years	.733	0.000*	
		10-12 years	.200	0.005*	
		13-16 years	-.067	0.972	
	7-9 years	Adult	-.733	0.000*	
		10-12 years	-.533	0.281	
		13-16 years	-.800	0.000*	
	10-12 years	Adult	-.200	0.005*	
		7-9 years	.533	0.281	
		13-16 years	-.267	0.307	
	13-16 years	Adult	.067	0.972	
		7-9 years	.800	0.000*	
		10-12 years	.267	0.307	
F1B4	Adult	7-9 years	1.000	0.000*	
		10-12 years	.267	0.005*	
		13-16 years	.000	1.000	
	7-9 years	Adult	-1.000	0.000*	
		10-12 years	-.733	0.323	
		13-16 years	-1.000	0.000*	
	10-12 years	Adult	-.267	0.005*	
		7-9 years	.733	0.323	
		13-16 years	-.267	0.549	
	13-16 years	Adult	.000	1.000	
		7-9 years	1.000	0.000*	
		10-12 years	.267	0.549	

As shown, significant differences between the adults and the children in the age groups of 7-9 and 10-12 years were observed in all tests. The difference between the adults and adolescents aged 13-16 was not remarkable, nor was the difference between the 7-9-year-old and the 10-12-year-old groups. However, the difference between the 7-9-year-old group and the 13-16-year-old group was significant. There was no notable difference between children aged 10-12 years and 13-16 years. Overall, the data from this study has shown that brain maturation can have a great effect on how auditory-visual stimuli interact.

## Discussion

This study examined the effects of auditory-visual maturation on SIFI behavioral tests in children, adolescents, and adults. Jiang et al. (2001) [19] reported multi-sensory pathways mature in the midbrain area before the cortex. Still, when they inactivate the cortico-collicular input, the ability to integrate auditory and visual multisensory information at the level of the superior colliculus nucleus was impaired [19]. Thus, although multi-sensory pathways mature later in the cortex surface than in other brain regions, the cortex itself controls how auditory-visual multisensory information is integrated into the midbrain. Therefore, the performance of multi-sensory processing is severely affected by any damage to the auditory-visual multi-sensory pathways at the cortex level [6]. For example, in autistic children, because the integration area of auditory-visual multi-sensory information is in the cortex (known as the posterior superior temporal gyrus), they have difficulty understanding and distinguishing spoken voices and cannot use lip reading [20].

The results of the SIFI test in F1B2, F1B3, and F1B4 tests showed that when the test was difficult, the 10-12 and 13-16-year-olds performances are better than the 7-9-year-old children. This large difference in more severe tests, such as the F1B4, is due to the complexity of the maturation of the auditory-visual multi-sensory pathways at the anterior ectosylvian sulcus cortex surface, which does not reach maturation, making it harder for younger aged children to perform the tasks [4]. Four factors cause 7-9-year-old children to score lower than adults. The first factor is the lack of maturity in the hearing pathways also causes the poor performance of 7-9-year-old children on more difficult tasks. For example, gap detection is one of the most important factors that give the child the ability to detect the time interval between two auditory stimuli [21]. The second factor is that as a person ages, they can detect shorter periods between two auditory stimuli, but this ability in 7-9-year-old children has not sufficiently matured [22]. The third factor is the non-sensory ones, such as attention and motivation, which should be considered. They strongly influence performance and are weaker in children than in adults, causing the child not to focus enough on the stimuli presented in complex tasks [23]. The fourth factor is the issue of distractors; at inferior age, distractibility increases, which can severely affect selective attention [24]. In the F1B4 test, the child is asked to focus on the screen and finally count the number of perceived flashes. Therefore, the first factor, inadequate attention, and concentration in 7-9-year-old children caused their performance to be significantly different from that of other age groups. Children need to discover the time difference between hearing stimuli to express the number of beeps accurately. However, children with poor gap detection ability are now unable to count the exact number of beeps. In this test, children were asked to indicate the number of perceived flashes; when provided 4 beeps simultaneously with the flash, the beeps became a distractor, causing the children to ignore the requested task. Ultimately, these factors led children (especially the 7-9-year-olds) to score lower on the SIFI test than adults.

Electrophysiological tests have examined the effect of combining auditory-visual information on multi-sensory processing. The results have shown that the scores on recorded tests increased with aging, which is consistent with the current study results [14]. It has also been shown that children about 6 years old and older can benefit from the integration of auditory-visual sensory for better speech understanding, especially in noisy environments. This benefit increases with age and indicates complete maturation of the auditory-visual multi-sensory pathways [25].

Delay or impairment in the maturation process of auditory-visual multi-sensory processing, effects on perception, decision-making, and even behavior resulting from auditory-visual sensory integration can be observed [6]. Therefore, any impairment of the auditory and visual system affects the maturation of auditory-visual multi-sensory processing. For example, Xu et al. (2014) [26] showed that constant noise affected the maturation of auditory pathways during the growth period of cats. Finally, by examining the maturity of the auditory-visual integration processes, a significant difference was observed compared to the controlled group. Therefore, any acquired or congenital damage in these two pathways can severely affect the maturation of auditory-visual multisensory processing [26]. However, when the damaged sense input is re-enabled, improvement of multi-sensory processing can be observed. For example, Gilley et al. (2010) [8] performed auditory-visual integration tests on children who have experienced several years of hearing impairment and compared the results with those with normal hearing. They found that reaction time was higher in children with cochlear implants than in children with normal hearing. Comparing two groups of cochlear implant patients, one group being early implanted and the other group being late implanted, they found that the reaction time of the early implanted children was faster than that of the late implanted children [8]. Therefore, it should keep in mind that the maturation of auditory-visual pathways is an adaptive process and can gradually continue to mature until full maturity is reached [27].

Although the results of auditory-visual integration tests were significantly different in children compared with adults, these differences decreased by age. Even if a child is temporarily deprived of hearing or vision, to compensate for this deprivation, the maturation of the auditory-visual multisensory processing can continue until it reaches final maturity.

## Conclusions

Based on the results obtained with the SIFI test, it is suggested to specify the normative data for each age group. The current results confirm that SIFI test results are different for individuals of different ages. Therefore, it is necessary to evaluate and analyze the results of different age groups with normative data to that specific age group. The results of studies will be strongly affected if the effects of age difference on audio-visual integration tests are ignored. In addition, the current study showed that the results of behavioral tests of audio-visual integration are completely consistent with electrophysiological tests. Therefore, if electrophysiological tests are not accessible, it is possible to use equivalent tests performed behaviorally.

## References

[1] Huttenlocher PR, Dabholkar AS (1997). Regional differences in synaptogenesis in human cerebral cortex. J Comp Neurol.

[2] Courchesne E, Chisum HJ, Townsend J (2000). Normal brain development and aging: quantitative analysis at in vivo MR imaging in healthy volunteers. Radiology.

[3] Zündorf IC, Lewald J, Karnath HO (2016). Testing the dual-pathway model for auditory processing in human cortex. Neuroimage.

[4] Wallace MT, Carriere BN, Perrault TJ Jr, Vaughan JW, Stein BE (2006). The development of cortical multisensory integration. J Neurosci.

[5] Arnold P, Köpsel A (1996). Lipreading, reading and memory of hearing and hearing-impaired children. Scand Audiol.

[6] Stein BE, Stanford TR, Rowland BA (2009). The neural basis of multisensory integration in the midbrain: its organization and maturation. Hear Res.

[7] Tibussek D, Meister H, Walger M, Foerst A, von WH (2002). Hearing loss in early infancy affects maturation of the auditory pathway. Dev Med Child Neurol.

[8] Gilley PM, Sharma A, Mitchell TV, Dorman MF (2010). The influence of a sensitive period for auditory-visual integration in children with cochlear implants. Restor Neurol Neurosci.

[9] Cuppini C, Ursino M, Magosso E, Ross LA, Foxe JJ, Molholm S (2017). A computational analysis of neural mechanisms underlying the maturation of multisensory speech integration in neurotypical children and those on the autism spectrum. Front Hum Neurosci.

[10] Wallace MT, Stein BE (2000). Onset of cross-modal synthesis in the neonatal superior colliculus is gated by the development of cortical influences. J Neurophysiol.

[11] Lippé S, Kovacevic N, McIntosh AR (2009). Differential maturation of brain signal complexity in the human auditory and visual system. Front Hum Neurosci.

[12] Kao CQ, McHaffie JG, Meredith MA, Stein BE (1994). Functional development of a central visual map in cat. J Neurophysiol.

[13] Brandwein AB, Foxe JJ, Russo NN, Altschuler TS, Gomes H, Molholm S (2011). The development of audiovisual multisensory integration across childhood and early adolescence: a high-density electrical mapping study. Cereb Cortex.

[14] Pillai R, Yathiraj A (2015). Auditory, visual, and auditory-visual processing performance in typically developing children: modality independence versus dependence. J Acoust Soc Am.

[15] Holm VA, Kunze LH (1969). Effect of chronic otitis media on language and speech development.. Pediatrics.

[16] Keane BP, Rosenthal O, Chun NH, Shams L (2010). Audiovisual integration in high functioning adults with autism. Res Autism Spectr Disord.

[17] Setti A, Stapleton J, Leahy D, Walsh C, Kenny RA, Newell FN (2014). Improving the efficiency of multisensory integration in older adults: audio-visual temporal discrimination training reduces susceptibility to the sound-induced flash illusion. Neuropsychologia.

[18] Shams L, Kamitani Y, Shimojo S (2002). Visual illusion induced by sound. Brain Res Cogn Brain Res.

[19] Jiang W, Wallace MT, Jiang H, Vaughan JW, Stein BE (2001). Two cortical areas mediate multisensory integration in superior colliculus neurons. J Neurophysiol.

[20] Dawes P, Bishop DV (2008). Maturation of visual and auditory temporal processing in school-aged children. J Speech Lang Hear Res.

[21] Snell KB (1997). Age-related changes in temporal gap detection. J Acoust Soc Am.

[22] Trehub SE, Schneider BA, Henderson JL (1995). Gap detection in infants, children, and adults. J Acoust Soc Am.

[23] Sutcliffe P, Bishop D (2005). Psychophysical design influences frequency discrimination performance in young children. J Exp Child Psychol.

[24] Robinson CW, Hawthorn AM, Rahman AN (2018). Developmental differences in filtering auditory and visual distractors during visual selective attention. Front Psychol.

[25] Barutchu A, Danaher J, Crewther SG, Innes-Brown H, Shivdasani MN, Paolini AG (2010). Audiovisual integration in noise by children and adults. J Exp Child Psychol.

[26] Xu J, Yu L, Rowland BA, Stanford TR, Stein BE (2014). Noise-rearing disrupts the maturation of multisensory integration. Eur J Neurosci.

[27] Mitchell TV, Maslin MT (2007). How vision matters for individuals with hearing loss. Int J Audiol.

